# Surface-enhanced Raman spectroscopic chemical imaging reveals distribution of pectin and its co-localization with xyloglucan inside onion epidermal cell wall

**DOI:** 10.1371/journal.pone.0250650

**Published:** 2021-05-05

**Authors:** Qing He, Jingyi Yang, Olga A. Zabotina, Chenxu Yu

**Affiliations:** 1 Department of Agricultural and Biosystems Engineering, Iowa State University, Ames, IA, United States of America; 2 Department of Molecular Biology, Biochemistry and Biophysics, Iowa State University, Ames, IA, United States of America; VIT University, INDIA

## Abstract

The primary plant cell wall is a complex matrix composed of interconnected polysaccharides including cellulose, hemicellulose, and pectin. Changes of this dynamic polysaccharide system play a critical role during plant cell development and differentiation. A better understanding of cell wall architectures can provide insight into the plant cell development. In this study, a Raman spectroscopic imaging approach was developed to visualize the distribution of plant cell wall polysaccharides. In this approach, Surface-enhanced Raman scattering (SERS through self-assembled silver nanoparticles) was combined with Raman labels (4-Aminothiophenol. 4ATP) and targeted enzymatic hydrolysis to improve the sensitivity, specificity, and throughput of the Raman imaging technique, and to reveal the distribution of pectin and its co-localization with xyloglucan inside onion epidermal cell (OEC) wall. This technique significantly decreased the required spectral acquisition time. The resulted Raman spectra showed a high Raman signal. The resulted Raman images successfully revealed and characterized the pectin distribution and its co-localization pattern with xyloglucan in OEC wall.

## Introduction

Polysaccharides are the main components of the plant cell wall. The complex network of polysaccharides consists of cellulose, hemicelluloses and pectin [1]. Cellulose is a linear unbranched polysaccharide formed of *β*-1-4-D-Glucose residuals. Hemicellulose is a group of heterogeneous polysaccharides that have *β*-(1,4)-linked backbones of various base units. Pectin is a group of complex polysaccharides formed with *α*-(1,4)-D-galacluronic acid residuals. The *α*-(1,4)-D-galacturonic acid is a unique monosaccharide due to its carboxylic group ester. Approximately 70% of pectin is comprised of *α*-(1,4)-D-galacturonic acid residues [2]. Some side chains of hemicelluloses and plant cell wall proteins contain a limited number of carboxylic groups as well. The main polysaccharides in the pectin group include homogalacturonan (HG), rhamnogalacturonan I (RG I), rhamnogalacturonan II (RG II), and xylogalacturonan (XGA) [1, 2]. Pectin fulfills many important plant biological functions, including growth, development, cell-cell adhesion, signaling, and enzyme modulation, etc [2]. The polysaccharides network defines the biomechanical properties of the plant cell wall [3], it is also the first line of defense that plant cells have against pathogen invasion [4]. However, our understanding of their structures, functions, and interactions with each other is still limited. The proposed plant cell models have been evolved in the past several decades [5–9]. Many studies paid particular interests to the interactions between pectin and xyloglucan, because the xyloglucan and pectin complexes would play important role in plant cell wall assembly and metabolism. Evidence has shown that there are covalent linkages between xyloglucan and pectin. An early plant cell wall model proposed the existence of covalent linkages between xyloglucan and RG-I based on the chromatographic co-elution of Xly residues and uronic acid residues [5]. Further evidence for xyloglucan-pectin complexes was provided through labelling ^14^C the pectic oligo-[^14^C]galactan chains, and observing the affinity change of ^14^C-labelled product after endo-1,4-glucanase treatment, which digests xyloglucan [10]. A later study showed that xyloglucan-pectin covalent complexes were found in a broad variety of cell-suspension cultures [7]. Although these studies provided insight into the pectin-xyloglucan complexes, more details of their distribution in the plant cell wall and their interactions with enzymes are still needed.

Several microscopic techniques have been employed to visualize the polysaccharides in the plant cell wall. The popular microscopic techniques including transmission electron microscopy (TEM) [11], confocal laser scanning microscopy (CLSM), fluorescence microscopy [12], and atomic force microscopy (AFM) [13]. None of them can yield detailed chemical images to reveal polysaccharide distribution and organization patterns in plant cell walls with micrometer resolution [14]. The confocal Raman microspectroscopy [15] is a non-invasive method, which could be utilized to obtain chemical images of polysaccharides in plant cell walls with micrometer resolution and beyond. The lignin and cellulose distribution in black spruce wood has been studied with Raman imaging [16]. The distribution of lignin and cellulose [17, 18] as well as phenolics and lipids [19] in Arabidopsis plant cell wall were studied with Raman imaging technique. The polysaccharides distribution and changes in plant cell wall during apple fruit development and senescence [20], fruit infected by Alternaria alternate [21], fruit lignification [22] have also been reported. However, Raman scattering is a weak phenomenon, long data acquisition time is often required to obtain Raman spectra with high signal-to-noise ratio(S/N). Moreover, the long spectral acquisition time could also lead to photo-damage to the samples under investigation [23, 24]. Surface-enhanced Raman scattering (SERS) provides a powerful solution to the low S/N problem [25]. In SERS, it has been shown that a signal enhancement of 10^4^−10^8^ could be achieved in comparison with normal Raman scattering [26, 27] for biological samples. To achieve such enhancement factor, Au or Ag nanostructures are commonly used as enhancers because their surface plasmons could resonate with electromagnetic wave in the same wavelength range as most of the commercially available lasers used for Raman excitation [28]. The effectiveness of SERS is distance-dependent. The strength of the resonated plasmonic electromagnetic field could decrease 10 times when the distance between the analytes and the SERS nanoenhancers increased by 2.8 nm [29]. Consequently, to achieve the highest enhancement factor, the target molecules need to be within a few nanometers distance from the nanoenhancers surface. Further enhancement to SERES signals could be generated by utilizing Raman labels in biological and molecular imaging [30–33], the Raman labels are molecules with large Raman scattering cross-section that would have strong Raman scattering signals when in resonance with the excitation laser. To ensure the Raman labels can be located close to the nanoenhancer, the Raman labels chosen usually contain sulfur or nitrogen atoms to be able to form covalent bonds with the nanoenhancers [34]. The combination of Raman labels and surface enhancement could achieve a level of enhancement of 10^14^ compared with the normal Raman scattering [29]. Different approaches have been explored to apply SERS combined with Raman labels in plant study. The distribution of a triplex Au-Ag-C core-shell NPs incorporated with the report molecule was monitored in a living plant leaf with SERS [35]. SERS image of report molecules attached to silver nanoparticles was utilized in onion plant cells to study the heterogeneous chemical structures and pH value inside the cells [15]. However, only limited number of research were conducted to characterize the cell wall polysaccharides distribution with SERS.

In most of these previous studies, the polysaccharide distribution was analyzed via Raman images constructed by the signature peaks of the polysaccharide itself, where each peak was correlated to a specific chemical bond in the polysaccharide. However, due to the similarity of the chemical bonds in most of the polysaccharides, this approach did not provide enough specificity to allow differentiation between different polysaccharides. Consequently, the Raman images constructed in these studies usually only differentiate polysaccharides such as cellulose from lignin [17, 18], but rarely among pectin molecules themselves.

In this study, we developed a new approach to improve the specificity of SERS imaging. We used a Raman tag (RT) 4-Aminothiophenol (4ATP) to label the *α*-(1,4)-D-glacluronic acid residuals on pectin backbone in onion epidermal cell (OEC) wall through covalent binding, and silver nanoparticles (AgNP) were layer-by-layer self-assembled onto the onion cell wall to form AgNP-4ATP conjugation which allowed chemical images to be created via the strong SERS signal of 4-ATP. The onion epidermal consists of a single layer cells surrounded by primary cell walls, which makes it a good system to be used for imaging. In addition, two enzymes, endo-polygalacturonase (EPG) and xyloglucan-specific endo-1,4-glucanase (XEG) were introduced to further improve the specificity. The Raman signal changes before and after the enzymatic hydrolyze are correlated to the enzymatic hydrolysis of the pectin and hemicellulose, respectively. Hence, the SERS images of the 4-ATP distribution in the cell walls were collected before and after EPG and XEG hydrolysis. The resulted changes in Raman signal (i.e., decrease) reflected the changes in the respective polysaccharides contents caused specifically by the enzymes. Through this analysis, the pectin distribution and its co-localization with xyloglucan could be characterized to reveal the sites of possible interaction between pectin and xyloglucan, which would help further our understanding of the cell wall structures.

## Materials and methods

### Chemical and biological materials

4-Aminothiophenol (4ATP, 97%), silver nitrate (AgNO_3_), sodium borohydride (NaBH_4_), Phosphate-buffered saline (PBS) 1× concentration, 1-ethyl-3-(-3-dimethylaminopropyl) carbodiimide hydrochloride (EDC), N-Hydroxysuccinimide (NHS) were all analytical grade, purchased from Sigma-Aldrich (St. Louis, MO) and used without further purification. The endo-polygalacturonanase M2 (*Aspergillus aculeatus*) enzyme was purchased from Megazyme (Bray, Ireland). The xyloglucan-specific endo-*β*-1,4-glucanase (XEG) was purchased from Novozymes (Copenhagen, Denmark). The silver enhancement reagent, R-Gent SE-LM electron microscopy grade silver enhancement reagent, was purchased from Aurion (Wageningen, The Netherlands). 18.2 M*Ω* E-pure water was used throughout the experiments.

### Preparation of onion skin to obtain single-layer epidermal cells

**Fig 1:**
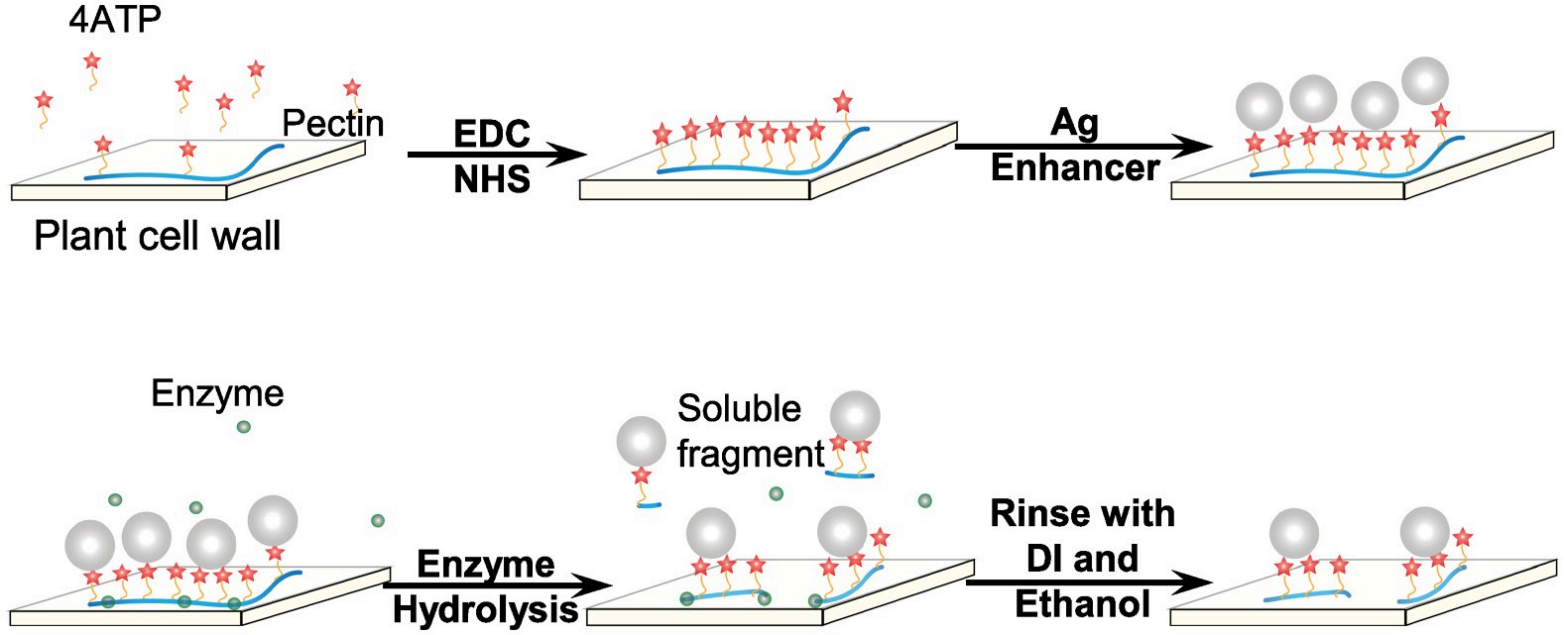
Experiment scheme. Layer-by-layer assemble of AgNP-4ATP-OEC and enzyme Hydrolysis of AgNP-4ATP-OEC.

To prepare the single-layer OEC samples, a white onion was cut into a rectangular piece, and the inner onion skin was peeled with a pair of tweezers. The 4ATP was then used as the RT to label the polysaccharides, which was subsequently conjugated to AgNPs for SERS imaging. The process to prepare the AgNP-4ATP conjugated OEC (AgNP-4ATP-OEC) was shown in Fig 1. Briefly, the peeled OEC was soaked in a freshly made mixture of 5 ml DI water, 5ml ethanol, 8 mg EDC, 6 mg NHS and 4 mg of 4ATP, and incubated for 30 minutes to prepare the 4ATP labeled OEC (4ATP-OEC). Another OEC sample was soaked in the solution with only 5 ml DI water, 5ml ethanol and 4 mg 4ATP and incubated for 30 minutes to prepare the 4ATP soaked OEC. The carbodiimide crosslinker chemistry with EDC-NHS induced peptide bond was well established [36, 37]. In our scheme, it assisted bond formation between amino group of the 4ATP and carboxylic group of the galacturonic acid in pectin. After the labeling process was finished, the samples were rinsed thoroughly with ethanol and DI water to wash off the unbound 4ATP. The AgNP-4ATP-OEC was prepared by drop coating the silver enhancement reagent onto the 4ATP-OEC to form the self-assembled silver nanoparticles layer for subsequent SERS imaging. In short, R-Gent SE-LM electron microscopy grade silver enhancement reagents, including the developer agent and enhancer agent, were mixed at equal parts at room temperature. The mixture was then applied onto the samples in a moisture chamber to form AgNPs *in situ*. Freshly peeled onion skin was directly reacted with the silver enhancement reagent to prepare the control (AgNP-OEC). After 30 minutes of incubation with silver enhancement reagents in the moisture chamber, all samples were extensively washed with DI water. The labeled onion samples were then cut into 5mm x 5mm pieces and fixed by waterproof tapes on gold slides for the following experiments. The 4ATP was mixed with AgNP to form the AgNP-4ATP complex. The AgNP was synthesized with NaBH_4_ as follow. A 5 x 10^-3^ M AgNO_3_ 10 mL was added drop by drop to ice-cold 2 x 10^-3^ M NaBH_4_ with vigorous stirring. The 4ATP-AgNP complex was formed by mixing 10 *μ*L 1 *μ*M 4ATP ethanol solution with 10 *μ*L freshly made AgNP and air dry on glass slides before Raman spectral acquisition. The normal Raman spectra of bulk 4ATP and SERS spectrum of 4ATP-AgNP complex were collected to demonstrate the 4ATP SERS behavior.

### Enzymatic hydrolysis of cell wall polysaccharides (pectin and xyloglucan)

The EPG was diluted to 25 U/mL, and the XEG was used at 0.5 mg/mL. All enzyme solutions were diluted with phosphate buffer (PBS, 10 mM, pH = 7.4). Series of enzyme-hydrolyzed AgNP-4ATP-OEC samples were prepared. For EPG hydrolysis, each sample was subject to 30 min hydrolysis. For the sequential hydrolysis, each sample was hydrolyzed with XEG for 30 minutes, followed by EPG for 30 minutes. Then, the samples were placed on a hot plate at 90°C for 20 minutes to denature the enzymes and terminate the hydrolysis reaction.

All hydrolysis was done at room temperature. After each treatment, samples were extensively rinsed with ethanol and DI water to remove unbound RTs and nanoparticles. All treatments were conducted in triplicate.

### SERS imaging

A dispersive Raman microscope (DXR, Thermo Scientific, Madison, WI) was used for Raman spectra acquisition with 532 nm excitation laser, 10mW laser power, 20x objectives, and 50 *μ*m pinhole. The laser exposure time and the number of accumulation were varied for different samples. The OMNICTM suite (Thermo Scientific, Waltham, MA, USA) was used for spectral acquisition and Raman mapping. For the Raman chemical image collection, each sample was scanned with a 2 *μ*m step size, and the pseudo-color image based on the RT 4ATP signature peak at 1573 cm^−1^ [38] was generated.

## Results

### Covalent binding of 4ATP onto the pectin and xyloglucan in OEC walls

An active Raman scatter, 4ATP [14], was used as the RT to label the polysaccharides in the OEC walls. The 4ATP contains a thiol group, which provides strong surface affinity to AgNP; an amino group that can form a peptide bond with carboxylic group on pectin molecules through the carbodiimide crosslinker chemistry and a benzene ring with high Raman cross-section. Approximately 70% of pectin is comprised of *α*-(1,4)-D-galacturonic acid as base unit [2]. Through the carbodiimide crosslinker chemistry, these carboxylic groups reacted with the EDC to form an activated ester named O-acylisourea. Since the EDC activated ester is unstable, NHS was introduced to convert the EDC activated ester into a relatively stable NHS-ester. The NHS-ester then crosslinked with the amine groups on 4ATP and form the covalent peptide bond [39].

To illustrate the Raman covalent bonding of 4ATP on OECs, the Raman spectra from the freshly peeled onion skin sample, the onion skin sample soaked in 4ATP solution (4ATP soaked-OEC) and the onion skin sample that covalently bonded by 4ATP (4ATP-OEC) were collected. All samples were placed on gold-coated slides and air-dried at room temperature to improve their Raman signals. The laser exposure time was set at 30 s for these tests, and 2 accumulations were acquired to improve the S/N. However, this exposure time was not suitable for Raman imaging, as the sample could be overheated or burned due to the long exposure to the laser [23, 24].

**Fig 2:**
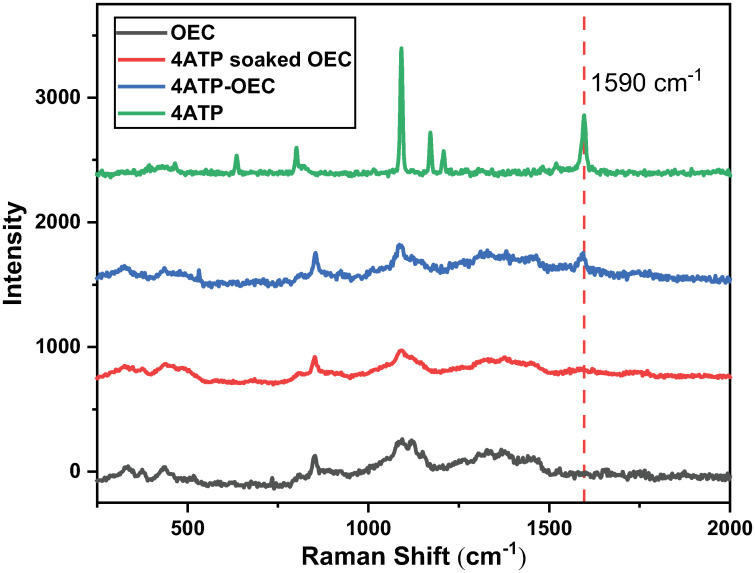
The Raman spectra of samples with different Raman tag binding methods. The Raman spectra collected from OEC, 4ATP soaked OEC, 4ATP-OEC, and bulk 4ATP powder.

As shown in Fig 2, the Raman spectra of these three samples are similar, indicating not much loss of polysaccharides due to the 4ATP binding procedure. The spectra collected from 4ATP-soaked onion showed nearly no 4ATP signature peaks, suggesting that no noticeable 4ATP-OEC binding occurred without carbodiimide crosslinker chemistry being in place. In comparison, the Raman spectrum collected from the ATP-OEC shows a clear 4ATP signature peak at 1590 cm^−1^. Clearly, 4ATPs were covalently bound to the OECs in this case.

### Self-assembly of silver nanoparticles onto 4ATP-OEC

**Fig 3:**
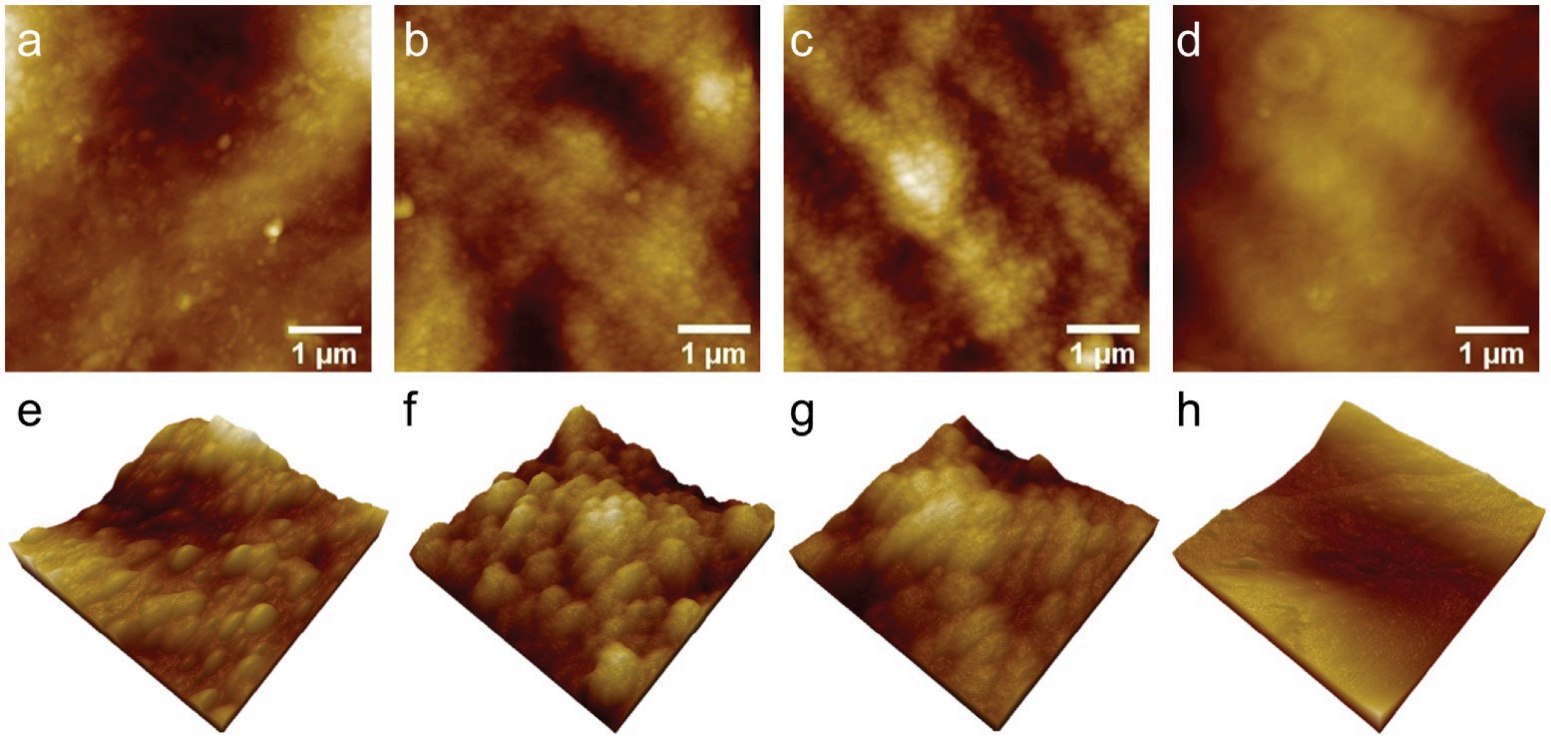
The AFM images of samples with different treatments. The AFM images of 5 *μ*m x 5 *μ*m areas (a)-(d) and zoomed-in 3D AFM images of 1 *μ*m x 1 *μ*m areas collected from AgNP-4ATP-OEC,AgNP-4ATP soaked-OEC, AgNP-OEC and freshly peeled OEC, respectively.

The self-assembly of silver nanoparticles that conjugated onto 4ATP labeled OECs to form AgNP-4ATP-OEC is depicted in Fig 1. The Raman tag 4ATP was covalently bond to the pectin, and the *in situ* formed AgNPs were self-assembled on to the 4ATP-OECs. The AgNPs were self-assembled onto the 4ATP-OECs through the thiol bond to form AgNP-4ATP-OECs. The 4ATP bonding AgNPs hence served as strong Raman surface enhancers to significantly enhance the SERS signal intensity of the 4ATP. The self-assembled AgNPs were characterized with AFM as shown in Fig 3. A 5 *μ*m x 5 *μ*m area of each sample was scanned with AFM for AgNP-4ATP-OEC, AgNP-4ATP soaked OEC, AgNP-OEC, OEC, respectively, as shown in Fig 3a–3d. The samples with *in situ* AgNP formation from the silver enhancer reagents showed rough surface with clustered nanostructures on the surface, while the OEC sample showed smooth surface. The zoomed-in AFM images of 1 *μ*m x 1 *μ*m areas were scanned for AgNP-4ATP-OEC, AgNP-4ATP soaked OEC, AgNP-OEC, OEC, respectively, as shown in Fig 3e–3h. As shown in Fig 3e, the AgNP on AgNP-4ATP-OEC were smooth hemispherical nanostructures scatter on the surface with a size range from 40 nm to 120 nm. The AgNP nanostructure formed a thin layer on AgNP-4ATP soaked OEC, AgNP-OEC surface. The AgNPs appears to be mounds with rough surfaces that are uniformly distributed with diameters ranged from 60 nm to 90 nm.

**Fig 4:**
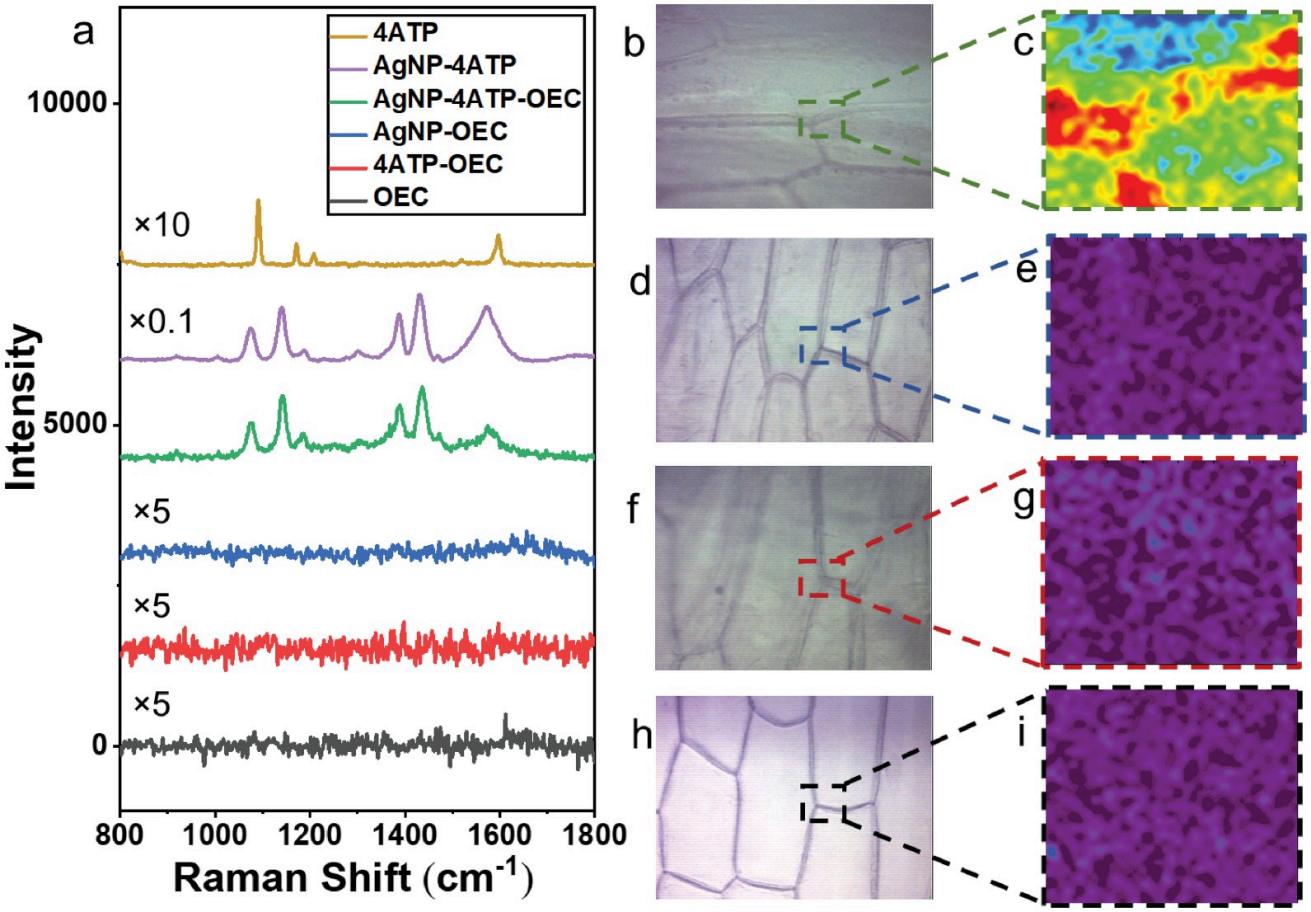
The Raman spectra and Raman images of samples with different treatments. The typical Raman spectra collected from bulk 4ATP, AgNP-4ATP complex, AgNP-4ATP-OEC, AgNP-OEC, 4ATP-OEC and freshly peeled OEC (a). The optical images and Raman images of scanned area created from AgNP-4ATP-OEC (b),(c), AgNP-OEC (d), (e), 4ATP-OEC (f), (g) freshly peeled OEC (h), (i), respectively. The Raman images were created with the 4ATP-AgNP complex signature peak at 1573 cm^−1^. Only the AgNP-4ATP-OEC samples produced images with good resolution.

The 4ATP SERS behavior was demonstrated in Fig 4a. The normal Raman spectrum of bulk 4ATP, and SERS spectrum of 4ATP-AgNP complex on glass slides were collected. The 4ATP-AgNP complex was formed by mixing 10 *μ*L 1 *μ*M 4ATP ethanol solution with 10 *μ*L freshly made AgNP and air dry on glass slides before Raman spectral acquisition. The laser exposure time was set at 1 s for these tests, and 3 accumulations were acquired to improve the S/N. The normal Raman spectrum of bulk 4ATP and SERS spectra of 4ATP absorbed on the AgNPs are significantly different. Two strong Raman bands at 1078 and 1589 cm^−1^ are observed in normal Raman spectra of bulk 4ATP, which are assigned to the vibration modes of *ν*CS(a_1_) and *ν*CSC(a_1_). The SERS spectrum of 4ATP-AgNPs complex shows multiple strongly enhanced bands at 1077, 1140, 1390 and 1440 cm^−1^ and multiple weakly enhanced bands at 1190, 1306, 1472, 1573 cm^−1^, which are commonly assigned to b_2_ modes of 4ATP. The selectively and strongly enhanced b_2_ bands of 4ATP could be explained by the charge transfer enhancement [40]. The SERS behavior of 4ATP is also observed in other plasmonic nanostructures [41]. The AgNP can strongly enhance the Raman signal of 4ATP by forming the AgNP-4ATP complex. When mixed with the AgNP, the diluted 4ATP can show a 100 times stronger Raman signal than bulk 4ATP. Thereby, through the 4ATP labeling and SERS signaling, the pectin distribution could be characterized from the AgNP-4ATP complex formed *in situ* on OEC with significantly shortened Raman acquisition time, from 30 seconds to less than 1 second for each Raman spectral acquisition. This is critically important for Raman mapping as the acquisition time for each spectrum dictated the total time needed to acquire one Raman image. In this study, Raman map of a 30 μm x 36 μm area was collected from each of the AgNP-4ATP-OEC, AgNP-OEC, 4ATP-OEC and fresh OEC samples. All samples were placed on the gold-coated slides, covered by cover glass and immersed in DI water to maintain the cell shape and integrity. The excitation laser focused at the top of the onion skin samples. The wet onion skin thickness range from 0.391 mm to 0.827 mm [42] which is much thicker than the confocal Raman spectroscopy’s focus length in Z direction [43]. Thereby the signal from Au substrate is minimal. The Au glass slides with 1000 Å au coating, is used to suppress the fluorescent from glass substrate. The exposure time for each spectral acquisition was set at 0.5 s, and 2 accumulations were acquired to improve the signal-to-noise ratio. The typical Raman spectrum of each sample is shown in Fig 4a. While all other samples showed no significant Raman signal, the AgNP-4ATP-OEC spectrum showed a strong SERS signal of the RT: 4ATP. The strongly enhanced Raman bands at 1140, 1380 and 1432 cm^−1^ indicate the covalent bonds between 4ATP and AgNP. The strongly enhanced bands at 1077, 1140, 1390 and 1440 cm^−1^ and weakly enhanced bands at 1190, 1306, 1472, 1573 cm^−1^ are all assigned to b2 mode of 4ATP. However, no SERS Raman bands of pectin could be identified from the spectrum collected from AgNP-4ATP-OEC. It could be explained by the 4ATP that is closely located at the hot spots on the surface of AgNP with high Raman cross-section, which enabled the strong enhancement of the 4ATP Raman signal. On the other hand, pectin is located further from the hot spots with a smaller Raman scattering cross-section, thereby the Raman signal of pectin is only weakly enhanced and difficult to identify in the AgNP-4ATP-OEC spectrum with the current Raman acquisition setting. Fig 4b, 4d, 4f and 4h showed the optical images of the AgNP-4ATP-OEC, AgNP-OEC, 4ATP-OEC and fresh OEC samples, respectively. The areas marked by the rectangular boxes were subject to SERS mapping.

Raman chemical images were constructed for the marked areas for each of the four samples based on the 4ATP SERS spectrum signature peak at 1573 cm^−1^, as shown in Fig 4c, 4e, 4g and 4i, respectively. The areas in red represent the areas with high Raman intensities and the areas in purple showed the areas with low Raman intensities. Since 4ATP molecules were covalently bound to pectin, the Raman map of 4ATP actually reflected the pectin distribution in the OECs. As shown in Fig 4b, the Raman peak intensity is the highest over the cell corner, middle lamella and the out layer of the cell wall that connected to the middle lamella which is consistent with the current understanding of plant cell wall pectin distribution in the plant cell wall [44]. However, the Raman images of the other three samples showed no structures that correlated to polysaccharide distribution in sampled areas. Clearly, the chemical labeling with 4ATP and the signal enhancement through SERS were both needed to create informative chemical images that reveal polysaccharide distribution inside the OEC wall. The Raman map of a 46 μm x 50 μm area was also collected from AgNP-4ATP soaked-OEC sample that placed on the gold-coated slides, covered by cover glass and immersed in DI water. The Raman spectra of bulk 4ATP, 4ATP-AgNP, AgNP-4ATP-OEC, AgNP-4ATP soaked-OEC were compared (S1 File). No significant Raman signal was observed in AgNP-4ATP soaked-OEC, which further proved that there is no 4ATP residual in 4ATP soaked-OEC after the rinsing with ethanol and DI water. It further validates that the carbodiimide crosslinker chemistry helped forming the covalent bonds between 4ATP and pectin in OEC. The Raman image of AgNP-4ATP soaked-OEC was shown in S1 File, no structures that correlated to polysaccharide distribution in sampled area was shown in the image.

### Polysaccharides organization and co-localization revealed by enzymatic hydrolysis-induced chemical changes

**Fig 5:**
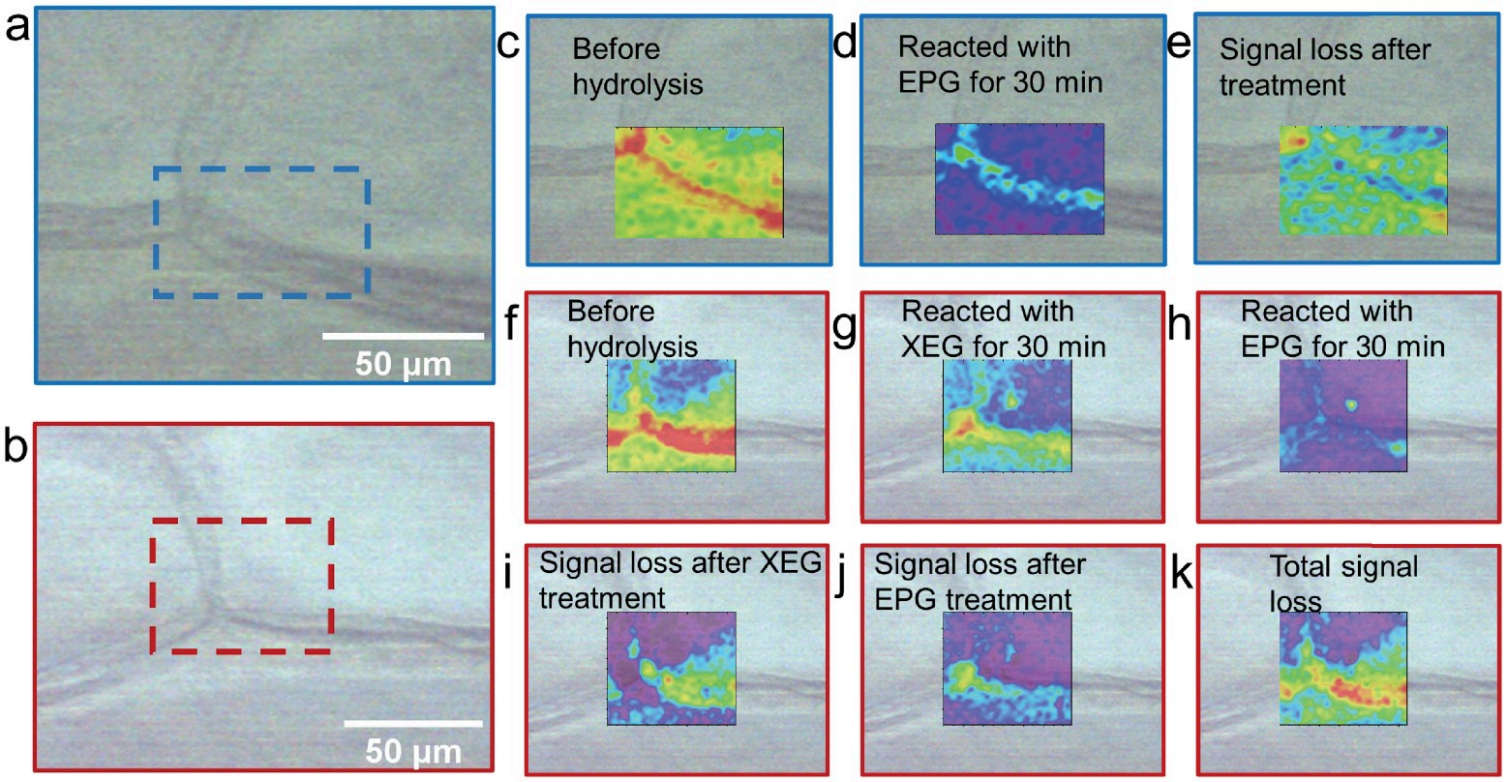
The Raman images of samples after enzymatic treatments. (a), (b) showed the optical image of sampled areas of samples that were treated with EPG enzyme only, and the sample treated with XEG and EPG in sequence, respectively. (c), (d) showed the Raman images of the sample before and after the EPG treatment. (e) shows the Raman image of Raman intensity differences before and after the EPG treatment. (f), (g), (h) showed the Raman image of the sampled area before the treatments, after XEG treatment, and after EPG treatment, respectively. (i), (j), (k) showed the Raman intensities change before and after XEG treatment, before and after EPG treatment, and the total intensity change after XEG and EPG treatment. All the Raman images were constructed based on the 4ATP signature peak at 1573 cm^−1^.

The Raman images of AgNP-4ATP-OEC samples before and after enzymatic hydrolysis reactions were constructed to illustrate the polysaccharides distribution and the effect of enzymatic treatment in the OEC wall. The optical microscopic images of the sample that was treated with EPG and the sample is shown in Fig 5a. The areas marked by blue rectangular is the area where the Raman images collected. A 40 *μ*m x 66 *μ*m area on EPG treated AgNP-4ATP-OEC was scanned before and after the treatment. The optical image of AgNP-4ATP-OEC that was treated with XEG and EPG sequence are shown in Fig 5b. The area marked by red rectangular is the areas where the Raman images were collected. A 58 *μ*m x 66 *μ*m area AgNP-4ATP-OEC treated with XEG and EPG sequence was scanned before and after the treatment.

The overlapping images of constructed Raman images and the optical image of the AgNP-4ATP-OEC sample before and after EGP treatment are shown in Fig 5c and 5d. The 4ATP signature peak intensity at 1573 cm^−1^ was used to generate Raman images. Fig 5c shows the Raman image before the EPG hydrolysis, Fig 5c shows the Raman image after 30 minutes of treatment with EPG, the areas in red represent the areas with higher Raman intensities and the areas in purple showed the areas with lower Raman intensities. As shown in the figures, the peak intensity decreased significantly after the sample reacted with EPG. Fig 5e shows the Raman intensities differences before and after hydrolysis. As shown in the figure, the Raman intensity decrease at the cell corner is significantly higher than other areas, the out layer of the cell wall that connects to the middle lamella and middle lamella also showed a high Raman intensity decrease.

To further reveal co-localization of a key hemicellulose, xyloglucan, with pectin, the AgNP-4ATP-OEC sample was subject to a sequential treatment of XEG and EPG. The results are shown in Fig 5f–5k. The Raman images are constructed based on the 4ATP signature peak at 1573 cm^−1^. The Raman image obtained before the enzymatic treatments are shown in Fig 5f. The sample was then treated with XEG for 30 minutes and the Raman image collected after the XEG treatment is shown in Fig 5g. The hydrolysis reaction with XEG enzyme results in the Raman peak intensity decrease. The sample treated with the XEG was then treated with EPG for 30 minutes, and a Raman image was collected and shown in Fig 5h. Clearly, the sample treated with XEG/EPG combined shows a much greater Raman intensity decrease compared to the sample only treated EPG. The changes in 4ATP signal which are tied to the hydrolysis of pectin and xyloglucan were reflected in the pseudo color images shown in Fig 5i–5k, with respect to Raman intensities differences before and after XEG hydrolysis, before and after EPG hydrolysis, and the overall decrease after the XEG and EPG treatment combined. As shown in Fig 5i, after the XEG hydrolysis, the highest Raman intensity decrease happened over the out layer of the cell wall that connects to the middle lamella area, while no Raman intensity decrease could be observed over the cell corner. It is also shown by Fig 5j that after treated by XEG, the EPG treatment caused the Raman intensity to significantly decrease at the cell corner, where from the earlier experiment it was already known that these areas have high pectin. There are also Raman intensity change at the middle lamella and the out layer of the cell wall that connects to the middle lamella area, as shown in Fig 5j. The overall Raman intensity change is shown in Fig 5k. Compared to the sample that was only treated by EPG, the sample that was first treated with XEG and then treated with EPG shows a higher Raman intensity decrease at the middle lamella area.

## Discussion

The polysaccharides distribution and their degradation by exogenously applied specific glycosyl hydrolases in the OEC wall were illustrated by the Raman images of different AgNP-4ATP-OEC samples before and after enzymatic hydrolysis reactions. The differences between the sample before and after treated with EPG would reveal where pectins are more abundant in the cell wall. The main polysaccharides in the pectin group include homogalacturonan (HG), rhamnogalacturonan I (RG I) and rhamnogalacturonan II (RG II) [1, 2]. The EPG hydrolysis can specifically hydrolyze the *α*-1,4-polygalacturonic acid backbone of HG and RG-II. After the sample treated with EPG, the peak intensity decreased significantly. The peak intensity decrease indicated that the pectin backbone in the OEC wall was hydrolyzed, and the soluble fragments containing the *α*-1,4-polygalacturonic residues were released into the solution, resulting in the decline of the *in situ* SERS signal. The differences in Raman intensities before and after EPG hydrolysis showed the Raman signal decrease at the cell corner and middle lamella between two cells was significantly higher than in other areas. The intercellular space over the cell corner mainly consists of low or partially methyl-esterified HG [45, 46], and the HG over middle lamella is partially esterified [47, 48]. HG is a linear polymer of alpha-1,4-polygalacturonic acid [1]. The carboxylic group rich backbone of HG allows a higher density of tagged AgNP-4ATP conjugation, and the linear molecular conformation allows better accessibility for EPG hydrolysis, which explains the higher Raman signal decrease at these areas. Additionally, the out layer of the cell wall that connects to the middle lamella also shows a high Raman intensity decrease, indicating that these are the locations of higher pectin accumulation within the cell wall.

The sequential treatment of XEG and EPG of the AgNP-4ATP-OEC sample further reveals the co-localization of xyloglucan, one of the main hemicellulose polysaccharide, with pectin. Some xyloglucans side chains cross-link with pectin, most likely RG-I [5, 6, 49, 50], to form a pectin-xyloglucan complex, which is possibly present in all primary cell walls [10]. A wide variety of angiosperm cell-suspension cultures show 30% to 70% of xyloglucan is associated with the forming of xyloglucan-pectin complexes [7]. Xyloglucan, cellulose, and pectin can further form an integrated complex as the load-bearing structure in the primary plant cell wall [1]. However, the distribution of co-localized pectin and xyloglucan is still unknown. The XEG treatment would break xyloglucan exposing more pectin for EPG hydrolysis. It was reasoned that differences between XEG/EPG treated samples and EPG only treated samples would reveal where co-localization of pectin and xyloglucan is more abundant in the cell wall. The hydrolysis reaction with XEG enzyme results in the Raman peak intensity decrease. This intensity decreases could be explained by the hydrolysis of the xyloglucan increases accessibility of pectin molecules to plant EPG localized in the cell wall. The sample reacted with the XEG was then reacted with EPG for 30 minutes. Clearly, the sample treated with XEG/EPG combined shows a much greater Raman intensity decrease compared to the sample treated with EPG only. This suggested the co-localization and interaction between pectin and xyloglucan in those specific locations within the onion cell wall. When the sample was treated only by EPG, the co-localized xyloglucan limited the accessibility of the enzyme to the pectin. However, when the sample was treated by the XEG first, the xyloglucan that co-localized with the pectin was hydrolyzed by the XEG and released to the media, and the exposed pectin molecules could be better exposed to the subsequent EPG hydrolysis.

The changes in 4ATP signal due to the hydrolysis of pectin and xyloglucan are presented as the pseudo color images reflecting the differences in Raman signal intensities observed before and after XEG hydrolysis, before and after EPG hydrolysis, and the overall decrease after the XEG and EPG combined treatment.

After only XEG hydrolysis, the highest Raman intensity decrease was observed in the outer layer of the cell walls closest to the middle lamella area, suggesting that there are areas with higher xyloglucan content co-localize with pectin. The interaction between pectin and xyloglucan plays a role in the cell to cell adhesion. Since the cellulose microfibrils are largely absent from the middle lamella, the cell to cell adhesion is mainly achieved by the pectins within the middle lamella and their interactions with the pectin, hemicellulose, and cellulose in the adjacent primary cell wall [51]. Specifically, the RG-I and/or RG-II side chains of covalently bonded RG-I, HG, and RG-II in the middle lamella are covalently bonded or hydrogen-bonded with the pectin, xyloglucan and cellulose in the adjacent primary cell wall [10, 52–55]. The highest Raman intensity decrease observed in the outer layer of the cell walls the closest to the middle lamella reflects the extensive interaction of xyloglucan with pectin at these areas. It provides evidence for pectin and xyloglucan’s role in the cell to cell adhesion. There is little Raman signal decrease over part of the cell surface area, suggesting these areas have limited pectin xyloglucan interaction, or/and firm pectin and cellulose association. No Raman signal decrease over the cell corner area could be observed, which could be explained by the absence of xyloglucan. After pretreatment with XEG, sequential treatment with EPG was conducted. The sequential EPG treatment caused the Raman intensity to significantly decrease at the cell corner, the out layer of the cell wall that connects to the middle lamella area and middle lamella area, the locations of higher accumulation of pectins according to our results shown in Fig 5g, whereas no much change was observed at the cell inner surface. When compared to the sample that was treated with EPG only, the sample treated sequentially with XEG and then with EPG shows higher overall Raman intensity decrease at the primary cell wall outer surface that is adjacent to middle lamella area and the middle lamella, indicating that these areas are where co-localization and interaction between xyloglucan and pectin occur the most. Most likely, xyloglucan bundled up with pectin at these areas limited access of pectin to hydrolysis by EPG. Current data based on immunolabelling gives contradictory conclusions about xyloglucan distribution in the middle lamella. Some studies showed that xyloglucan was distributed throughout the cell wall and middle lamella [56], while others reported that the xyloglucan was either absent or rarely exist in the middle lamella area [57]. The lack of xyloglucan in the middle lamella could be explained by either the lack of xyloglucan or the existence of different kinds of xyloglucan at the middle lamella, thereby the specificity of the immunolabelling could be limited by the specificity of the antibodies. With our method, we visualized the xyloglucan that co-localized with the pectin in the plant cell wall and middle lamella with the specificity provided by the enzymes instead of antibodies. However, since the areas of the middle lamella is too narrow, at this point it is difficult to conclude the presence of xyloglucan specifically in that area. In the future, this problem could be resolved by further improving the resolution of the Raman imaging through decrease the scanning step size.

## Conclusion

In this study, a novel Raman chemical imaging method was developed to employ SERS imaging to characterize polysaccharide distribution and organization in onion epidermal cell wall. The utilization of this new method to visualize polysaccharide distribution was assisted by the application of highly specific glycosyl hydrolases to degrade hemicellulose and pectin molecules in cell wall *in situ*. Using this combined approach, the colocalization patterns of pectin and xyloglucan in onion epidermal cell wall were characterized. This method enables SERS imaging to be applied to cell wall polysaccharides which are not sensitive to SERS methods in general. The Raman images showed that the polysaccharide distribution is consistent with the current understanding of plant cell walls organization. The Raman images indicated that there are interactions between pectin and xyloglucan at the outer layer of the cell wall that connects to the middle lamella area, and in the middle lamella area, while no significant pectin and xyloglucan interaction could be observed at the cell corner and in the cell inner surface. We propose that by expanding the number of glycosyl hydrolases with diverse specificity this approach can also be applied to analyze the distribution of the different pectin molecules, including the HG, RG I and RG II. The resolution of the Raman image could be further improved by using near field microscopy to get to sub-micron or even nanometer level. The method can potentially be used to generate the detailed chemical image to reveal polysaccharide distribution and interconnected patterns in the plant cell wall to increase our knowledge about cell wall molecular organization and improve existing cell wall structural models.

## Supporting information

S1 File The Raman spectra and Raman image of AgNP-4ATP soaked-OEC. (PDF)
