# Supplementary material for: Surface-enhanced Raman spectroscopic chemical imaging reveals distribution of pectin and its co-localization with xyloglucan inside onion epidermal cell wall
Source: PLoS One. 2021 May 5;16(5):e0250650. doi: 10.1371/journal.pone.0250650 (PMC8099099; doi:10.1371/journal.pone.0250650)
Supplement: S1 File — (PDF) [file pone.0250650.s001.pdf]

Surface-enhanced Raman spectroscopic chemical imaging reveals distribution of pectin and its co-localization with xyloglucan inside onion epidermal cell wall

Qing He[1,¤], Jingyi Yang[1], Olga A. Zabotina[2], Chenxu Yu[1*]

1 Department of Agricultural and Biosystems Engineering, Iowa State University, Ames, IA, United States
2 Department of Molecular Biology, Biochemistry and Biophysics, Iowa State University, Ames, United States
¤Current Address: Department of Electrical and Computer Engineering, University of Oklahoma, Norman, OK, United States

* Corresponding author
E-mail: chenxuyu@iastate.edu (CY)

Raman spectrum and Raman image of AgNP-4ATP soaked-OEC

The Raman map of a 46 µm x 50 µm area was collected from AgNP-4ATP soaked-OEC sample that placed on the gold-coated slide, covered by cover glass and immersed in DI water. The Raman image was collected with confocal Raman spectroscopy (Renishaw inVia, Renishaw, Wotton-under-Edge, UK) with 532 nm excitation laser, 50x objectives. The laser exposure time was set to be 0.5 second, 2 accumulations collected for each spectral acquisition. The Raman spectra of bulk 4ATP, 4ATP-AgNP complex, AgNP-4ATP-OEC, AgNP-4ATP soaked-OEC were compared as shown in Fig S1. The AgNP-4ATP soaked-OEC spectra was multiplied by 5, the bulk 4ATP was multiplied by 10, 4ATP-AgNP complex was multiplied by 0.1. The AgNP-4ATP soaked-OEC spectrum shown low Raman intensity. Moreover, no significant Raman signal was observed in AgNP-4ATP soaked-OEC, which further proved that there is no 4ATP residual in 4ATP soaked-OEC after the rinsing with ethanol and DI water. It further validates that the carbodiimide crosslinker chemistry helped forming the covalent bonds between 4ATP and pectin in OEC.

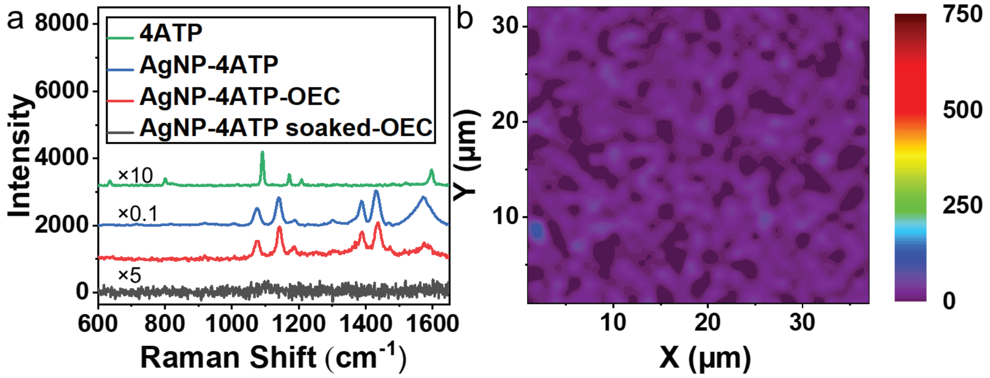

Figure S 1. The Raman spectra and Raman image of AgNP-4ATP soaked-OEC. The typical Raman spectra collected from bulk 4ATP, AgNP-4ATP complex, AgNP-4ATP-OEC and AgNP-4ATP soaked-OEC (a). The Raman images of scanned area created from AgNP-4ATP soaked-OEC (b).
